# A Comparison of the Antinociceptive Properties of SJP-005 and Morphine in Rats

**DOI:** 10.3390/pharmaceutics13020243

**Published:** 2021-02-10

**Authors:** Joris C Verster, Andrew Scholey, Thomas A Dahl, Jacqueline M Iversen

**Affiliations:** 1 Division of Pharmacology, Utrecht Institute for Pharmaceutical Sciences (UIPS), Utrecht University, 3584CG Utrecht, The Netherlands; j.c.verster@uu.nl; 2 Centre for Human Psychopharmacology, Swinburne University, Melbourne, VIC 3122, Australia; andrew@scholeylab.com; 3 Sen-Jam Pharmaceutical, 223 Wall St., #130, Huntington, NY 11743, USA; tadahl@sen-jam.com

**Keywords:** pain, analgesic properties, opioid withdrawal, opioid dependence, opioid use disorder, treatment, SJP-005, TRL4 inhibitor, PPAR-γ

## Abstract

SJP-005 (a combination of ketotifen and ibuprofen) is being developed as a potential treatment for pain and for opioid use disorder. It is therefore important to investigate the potential antinociceptive properties of SJP-005. Two studies were conducted to evaluate the potential effects of SJP-005 in rats. Study 1 applied the von Frey test to examine the antinociceptive effect of morphine with and without SJP-005 in adjuvant-induced hypersensitivity to tactile stimulation. In a double-blind, between-groups design, groups of rats (*n* = 10 each) received morphine at 3, 10, or 30 mg/kg bodyweight (bw) (subcutaneous injection) with or without SJP-005 (oral). Mechanic allodynia and paw volume were assessed before and after treatment. Study 2 utilized the hot plate test. Using a crossover design, groups of rats (*n* = 10 each) received either morphine at 3, 10, or 30 mg/kg bw (subcutaneous injection) preceded by oral administration of placebo (Week 1) or SJP-005 (Week 2). In Study 1, in the von Frey up-and-down test, Δ paw withdrawal responses in Group 1 (3 mg/kg bw morphine) were significantly lower compared to those in Group 4 (3 mg/kg bw morphine plus SJP-005), whereas the differences in Δ paw withdrawal between Group 2 and Group 5 (10 mg/kg bw morphine with and without SJP-005) and between Group 3 and Group 6 (10 mg/kg bw morphine with and without SJP-005) did not reach statistical difference. Trendline analysis of the dose–response relationship for the morphine + placebo groups and morphine + SJP-005 groups revealed no significant differences in the intercepts and slopes. In Study 2, no significant differences were observed on hot plate performance between morphine and morphine in combination with SJP-005. In conclusion, the findings in the von Frey up-and-down test (Study 1) suggest that animals can withstand higher levels of painful stimuli when SJP-005 is co-administered. This may also suggest a possible opioid sparing effect. However, in the hot plate test (Study 2), animals did not respond more adaptively to stronger painful stimuli after co-administering SJP-005. These observations warrant further investigation of the antinociceptive properties of SJP-005.

## 1. Introduction

Opioid withdrawal symptoms comprise a constellation of “flu-like” symptoms, including feeling sick, muscle spasms/twitching, feeling of coldness, heart pounding, aches and pains, insomnia/trouble sleeping, sweating, restlessness, gastrointestinal complaints, tremor, and anxiety or irritability. While opioid drugs such as morphine and oxycodone are effective for reducing acute pain and as anesthesia during surgery, these drugs also have high potential for abuse and dependency. This may lead to prolonged opioid use which may ultimately result in opioid use disorder.

In the U.S., opioid pain medication use has resulted in the opioid crisis [1,2]. Opioid dependency and misuse has developed into a serious, ongoing public health problem and, in 2017, resulted in the opioid-use-related deaths of over 47,600 Americans [3]. Also, in Europe, the use of prescription opioid pain medication is prevalent, with an estimated 22.8 million users in 2016. About 500,000 patients suffered then from opioid dependency [4], and these numbers have not attenuated [5,6]. It is therefore essential that effective and safe treatments are developed to aid in the treatment of pain, reduce opioid use, and prevent or treat opioid use disorder. 

Current treatments for opioid withdrawal, i.e., clonidine and lofexidine, have no relevant effect on pain. As pain and opioid abuse co-occur frequently [7], adjuvant treatments are prescribed to counteract the pain complaints of patients undergoing opioid withdrawal treatment. Because the intensity and duration of withdrawal symptoms can be severe, ceasing opioid use has proven difficult utilizing the current treatment regimens. That is, patients often fail to comply with treatment, and relapse rates have been reported in 32–70% in patients within six months after treatment initiation and 72 to 88% of patients after 12–36 months [8,9].

SJP-005, a new product that is being developed as a pain treatment and for opioid use disorder, is a combination product of an antihistamine drug with Toll-like receptor 4 (TLR4) inhibition properties (ketotifen), and a non-steroidal anti-inflammatory drug (NSAID) which also possesses peroxisome proliferator-activated gamma receptor (PPAR-γ) agonist activity (ibuprofen) [10,11]. Previous research in rodents [12] suggests that SJP-005 is effective in significantly reducing opioid withdrawal signs after cessation of morphine treatment. In three studies, rats were treated twice daily for 19 days with morphine. Treatment with morphine was ceased on Day 19, and withdrawal signs were recorded on the day of discontinuation (early phase) and on Days 20–30 (late phase). SJP-005 was administered twice daily, starting four or two days before or directly after morphine cessation. Overall, the three studies found that after administering SJP-005, lesser withdrawal signs were observed compared to placebo (spontaneous withdrawal). This effect was significant in the late phase of withdrawal (2–12 days after withdrawal) when SJP-005 was administered two days before morphine cessation. In addition, the number of days it took to observe zero withdrawal signs was significantly shorter when SJP-005 was administered two days before morphine cessation [12]. Taken together, these findings suggest that SJP-005 is a suitable candidate drug to be used for opioid withdrawal, and it deserves further investigation. 

While ibuprofen is an analgesic drug, research into possible antinociceptive properties of ketotifen is scarce, and in this context, the drugs have not been investigated in combination. A literature search identified one study examining the antinociceptive properties of ketotifen. Using a hot plate test in mice [13], it was found that ketotifen (2, 4, or 8 mg/kg) caused dose-dependent antinociceptive effects after naltrexone-induced withdrawal from morphine treatment (10 mg/kg). Coadministration of ketotifen with morphine attenuated morphine tolerance and reduced withdrawal signs. 

In this article we present the results of two studies that investigated the possible antinociceptive properties of SJP-005. These were investigated using two validated methodologies to evaluate pain in rodents, i.e., the von Frey test to assess inflammation-related pain (Study 1) and the hot plate test to assess centrally acting pain (Study 2) [14]. It was hypothesized that SJP-005, containing ibuprofen, a prostaglandin inhibitor, would reduce inflammation and, therefore, inflammation-related pain. Ketotifen’s role, as a mast cell stabilizer or inhibitor, can also reduce the release of prostaglandins and a number of other inflammatory mediators, including histamine. Given this, in Study 1 where inflammatory pain was induced in rats by the intraplantar injection of complete Freund’s adjuvant (CFA) and assessed by paw withdrawal, it was expected that SJP-005 could modify the anti-inflammatory pain response. In Study 2, the hot plate test, a centrally acting pain response was measured. Morphine is very effective in reducing centrally acting pain, whereas inhibitors of prostaglandin and histamine have not been found to be very effective in reducing this type of pain. Therefore, in Study 2, no relevant effect of SJP-005 was expected. Finally, the concept of “opioid sparing” was explored. Intravenous ibuprofen has that designation, based on the lower morphine requirements in post-surgery patients. It was hypothesized that the antinociceptive effects of SJP-005 in Study 1 might be accompanied by such an opioid sparing effect.

## 2. Materials and Methods

Two studies were conducted to compare the antinociceptive effects of SJP-005 and morphine. The two studies were funded by Sen-Jam Pharmaceutical and conducted by Calvert Laboratories, Inc. with ethics approval granted by the Calvert Institutional Animal Care and Use Committee (IACUC). The study protocols 1270RS128.001 (Study 1) and 0241RS128.001 (Study 2) were approved on October 18, 2018. Sprague Dawley rats were experimentally naïve at the start of the studies and were obtained from Charles River Laboratories, Raleigh, NC, USA. The animals were all males and were, on average, 7 weeks old (range 5–9 weeks) at the time of first dosing.

### 2.1. Housing and Handling of Animals

The treatment of Sprague Dawley rats used in these studies was in accordance with Calvert standard operation procedures, which adhere to the regulations outlined in the US Department of Agriculture Animal Welfare Act and the conditions specified in the Guide for the Care and Use of Laboratory Animals (Institute for Laboratory Animal Research (ILAR) publication, National Research Council, 2011, The National Academies Press). Animals were group housed in compliance with the National Research Council’s Guide for the Care and Use of Laboratory Animals. For identification purposes, animals were ear-tagged and color coded. Assessments of pain and distress and the non-use of pain alleviating medication during the morphine withdrawal experiments were in accordance with the Criteria for Assessing Pain and Distress in Laboratory Animals. Under controlled circumstances (12 h light/dark cycle, 20–26 Celsius, and 30–70% humidity), animals were kept in a separate room specifically dedicated to carrying out the studies and had access to a Certified Rodent Diet (TEKLAD) or equivalent and water ad libitum. Animals were acclimated to the room for 7 to 8 days prior to first dosing. During this period, animals were monitored and were replaced in case of observed signs of infectious disease. Rats with a normal body weight and no adverse clinical signs were eligible to participate in the study and were randomly assigned to one of the treatment groups. Treatment administration and all assessments were conducted in the same room. Within each study, the tests were conducted by the same investigator. The studies were not blinded (i.e., investigators were aware of the administered treatments).

### 2.2. Study 1

Study 1 (1270RS128.001) was designed to evaluate the potential effects of SJP-005 (1 mg/kg bodyweight (bw) ketotifen and 30 mg/kg bw ibuprofen) on the antinociceptive effect of morphine in adjuvant-induced hypersensitivity to tactile stimulation in rats. To this extent, an animal model frequently used to study pain associated with inflammation was applied: the subcutaneous injection of complete Freund’s adjuvant (CFA) followed by assessment of the paw withdrawal response using the von Frey’s up-and-down method (i.e., determining the mechanical force required to elicit a paw withdrawal response in 50% of animals) [14,15,16].

Seventy Sprague Dawley rats were tested. Three groups of *n* = 10 rats each received an oral dose of carboxymethylcellulose (placebo), followed 90 min later by a subcutaneous morphine injection of 3 mg/kg bw (Group 1), 10 mg/kg bw (Group 2), or 30 mg/kg bw (Group 3). The four other groups of *n* = 10 rats each received an oral dose of SJP-005 (1 mg/kg ketotifen and 30 mg/kg bw ibuprofen), followed 90 min later by a subcutaneous morphine injection of 3 mg/kg bw (Group 4), 10 mg/kg bw (Group 5), or 30 mg/kg bw (Group 6), or a subcutaneous injection with saline (placebo, Group 7). Morphine sulphate was supplied by Spectrum (Gardena, CA, USA), and carboxymethylcellulose, ketotifen fumarate, and ibuprofen were supplied by Sigma (St. Louis, MO, USA).

Approximately 24 h before treatment administration, paw volume was assessed by water displacement/digital caliper and mechanical allodynia (i.e., central pain sensitization following normally non-painful, repetitive stimulation) was assessed using von Frey’s up-and-down method [13,14,15]. This test measures the minimal mechanical force (g) that is required to elicit a paw withdrawal response. Responses to filaments with subsequently increasing or decreasing mechanical force, i.e., up and down, were tested with variable mechanical force until the filament corresponding to the withdrawal threshold was determined. Typically, we started with a 1 or 2 g filament and continued until a withdrawal response occurred or 300 g force was reached. For the second and third tests, the filament below the one that produced a response in the previous test was used first. The median force producing a response, determined from three tests given over a 20-min period (10-min rest period between tests), was considered the withdrawal threshold.

After these assessments, complete Freund’s adjuvant (0.5 mg/mL) was injected into the plantar surface on the footpad of isoflurane-anesthetized rats. On the day of dosing, test drugs or vehicle were orally administered 90 min prior to morphine or saline treatment. Approximately 30 min thereafter, paw volume/thickness and mechanical allodynia were reassessed. Paw volume/thickness was expressed as the percent inhibition of paw edema (mL) and calculated as follows: ((mean paw edema [control] − mean paw edema [test])/(mean paw edema [control]) × 100).

Statistical analyses were conducted using SPSS, version 25 (Armonk, IBM Corp., New York, NY, USA). First, the paw withdrawal response and paw volume/thickness, prior to and after treatment, were computed and for each group separately compared using paired-sample *t*-tests. Second, a possible dose–response effect was evaluated by analysis of variance (ANOVA) for Groups 1–3 and Groups 4–6. Third, to determine a possible additive effect of SJP-005 to the antinociceptive effects of morphine, independent sample *t*-tests were used to compare Group 1 and Group 4 (3 mg/kg bw morphine, with or without SJP-005), Group 2 and Group 5 (10 mg/kg bw morphine, with or without SJP-005), and Group 3 and Group 6 (30 mg/kg bw morphine, with or without SJP-005). Differences between groups were considered statistically significant if *p* < 0.05. Trendlines of corresponding Δ paw withdrawal response across the morphine dosages (3, 10, and 30 mg/kg bw) of the groups with and without coadministration of SJP-005 were statistically compared using Statgraphics Centurion, version 19. The equation of the trendlines (*y* = *ax* + *b*) and the intercept and slope were determined and compared using ANOVA. Differences were considered statistically significant if *p* < 0.05.

### 2.3. Study 2

The purpose of Study 2 (protocol number 0241RS128.001) was to evaluate the effects of morphine and SJP-005 on pain response induced by a hot plate test in rats. Forty Sprague Dawley rats were tested in a crossover design, i.e., they served as their own controls. 

On test Day 1, the hot plate test was conducted pre-dose. Only those rats that displayed a reaction time of 15 s or less during this baseline assessment were included in the study. Rats were randomly allocated to one of four groups of *n* = 10 rats. Animals received a subcutaneous injection of saline (placebo, Group 1) or morphine at 3 mg/kg bw (Group 2), 10 mg/kg bw (Group 3), or 30 mg/kg bw (Group 4). Morphine sulphate was supplied by Spectrum, saline by Vedco, and ketotifen fumarate and ibuprofen by Sigma. Thirty minutes following dosing, rats again completed the hot plate test. On test Day 2, one week later, the animals received the same treatment as in Week 1, which was preceded (-90 min) by an oral dose of SJP-005 (1 mg/kg ketotifen and 30 mg/kg bw ibuprofen). Thirty minutes following dosing, the hot plate test was completed.

In the hot plate test [14,17], rats were sequentially placed on a model 39D Hot Plate Analgesia Meter, set for 55 ± 2 Celsius. Characteristic reactions to the heat stimulus (i.e., licking the forepaw, rapid fanning of a hind paw, or a sudden jump on the hot plate) were recorded. When any of these endpoints was displayed, the rat was removed from the hot plate. Elapsed time was measured using a stop watch, accurate to at least 1/10 of a second. If no response was shown, animals were removed from the hot plate after 30 s. The primary outcome measure was the time (in seconds) between placing of the rat on the hot plate and the display of the endpoint. 

Statistical analyses were conducted using SPSS, version 25 (Armonk, IBM Corp, New York, NY, USA). Pre- and post-dose reaction times were computed for both test days. The analgesic response, i.e., the mean increase in reaction time to the heat stimuli, was calculated for each group. The percentage analgesia was computed as follows: % analgesia = ([average response time after treatment/average response time with no treatment] − 1.0) × 100. To evaluate a possible additive effect of SJP-005 to the antinociceptive effects of morphine, an ANOVA, with two-sided post hoc Tukey’s honestly significant difference (HSD) tests for multiple comparisons, was applied. The same test was used to compare treatments with placebo. Differences between groups were considered statistically significant if *p* < 0.05.

### 2.4. Safety and Adverse Effects

Individual body weights were assessed daily, and adverse effects were monitored throughout the study. Animals were observed once daily during acclimation. They were then observed when they were put on study, and at the time of dosing and any data collection measurement. Adverse effects of pain or distress or any adverse clinical signs were monitored. If any clinical signs were observed, they were recorded. Animals displaying signs of pain or distress during the study were euthanized. Animals found dead or prematurely sacrificed for humane reasons were subjected to necropsy and abnormalities were recorded. Dropouts were not replaced. After completion of the study, all animals were euthanized by CO_2_ asphyxiation, and death was confirmed via thoracotomy.

## 3. Results

### 3.1. Study 1

All animals completed the experiment, and no adverse effects were observed throughout the study. The results are summarized in Table 1 and Table 2. Individual animal data are listed in Appendix A.

Paw volume/thickness significantly increased after dosing in all seven groups (*p* < 0.0001) (see Table 1), but no significant differences between the groups were found for edema (F_(6,63)_ = 1.931, *p* = 0.089). In addition, the pre-dose paw withdrawal response (see Table 2) did not significantly differ between the groups (F_(6,63)_ = 0.266, *p* = 0.951). These observations justify a direct comparison of the post-dose assessments of the seven groups.

Compared to pre-dose assessments, significant post-dose increases in paw withdrawal response were found for all groups, except after administering SJP-005 only, which showed a non-significant decrease in post-dose paw withdrawal (Group 7). Compared to pre-dose assessments, significant differences between the groups were observed post-dose (F_(6,63)_ = 21.29, *p* < 0.000): animals of Groups 2–6 (i.e., morphine with or without SJP-005), but not Group 1 (morphine 3 mg/kg bw), performed significantly better than SJP-005 alone (*p* < 0.0001).

Morphine alone (Groups 1, 2, and 3) produced a dose-dependent increase in mechanical force required to elicit the paw withdrawal response (F_(2,27)_= 21.58, *p* < 0.000). A similar, significant dose-dependent increase towards a plateau (F_(2,27)_= 6.09, *p* = 0.007) was observed when comparing the groups who received morphine in combination with SJP-005 (Groups 4, 5, and 6). No significant differences were found for the paired comparisons between Group 1 and Group 4, between Group 2 and Group 5, and between Group 3 and Group 6 (see Table 2).

**Figure 1:**
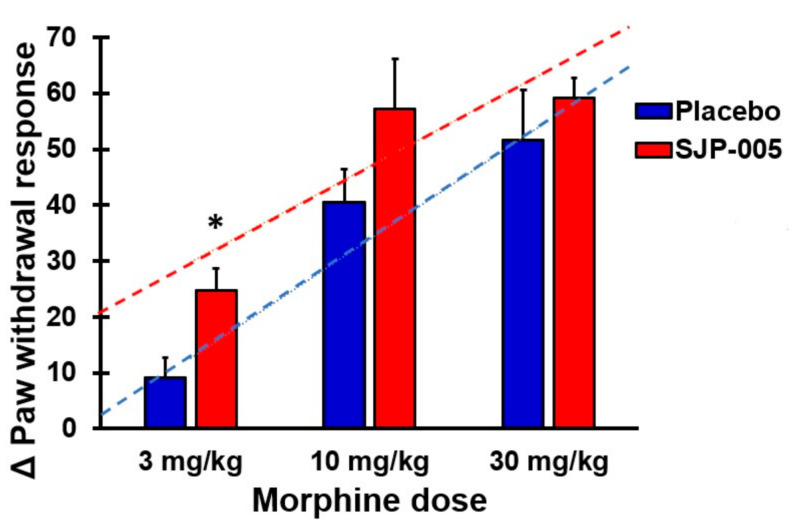
Paw withdrawal response (pre–post difference scores) after SJP-005 or placebo. The mean difference scores between post- and pre-dose assessments are shown. Error bars represent the standard error of the mean. Significant differences (*p* < 0.05) are indicated with *. Dashed lines represent the best-fitting linear trend lines for the dose–response relationship of morphine + placebo (blue color) and morphine + SJP-005 (red color). Each of the treatment groups consisted of 10 rats.

Figure 1 summarizes the difference scores on paw withdrawal (Δ, post-dose minus pre-dose) for Groups 1–6. Visual inspection of the data suggests a trend towards improvement in the paw withdrawal response in the groups treated with morphine in combination with SJP-005. In other words, animals seem to withstand higher levels of painful stimuli when SJP-005 is co-administered. Statistical analysis revealed that the Δ paw withdrawal responses in Group 1 (3 mg/kg bw morphine) were significantly lower (*t* = −2.928, *p* = 0.009) compared to those in Group 4 (3 mg/kg morphine plus SJP-005), whereas the differences in Δ paw withdrawal between Group 2 and Group 5 (10 mg/kg bw morphine with and without SJP-005) and between Group 3 and Group 6 (10 mg/kg bw morphine with and without SJP-005) were not statistically significant.

Regression analysis was conducted to compare the trendlines of morphine treatments with and without co-administration of SJP-005. Linear trendlines (see Figure 1) were computed for the morphine + placebo group (*y* = 1.35*x* + 14.4) and morphine + SJP-005 group (*y* = 1.01*x* + 32.4). ANOVA revealed that the intercepts and slopes of the trendlines did not significantly differ between the placebo and SJP-005 groups.

### 3.2. Study 2

In Study 2, one rat was found dead the day following the first treatment day. The cause of death was unknown. No adverse effects were observed throughout the study. The results are summarized in Table 3. Individual animal data are listed in Appendix B.

Compared to placebo, morphine produced dose-dependent increases in the mean reaction time to the heat stimulus (see Table 3). The increase in reaction time was statistically significant for morphine at 10 mg/kg bw and 30 mg/kg bw. Compared to morphine with placebo, morphine in combination with SJP-005 also produced dose-dependent increases in the mean reaction time to the heat stimulus. The difference in reaction time from placebo was statistically significant for the highest morphine dose in combination with SJP-005. Within-group comparisons revealed no statistically significant differences when morphine was administered alone (Week 1) or in combination with SJP-005 (Week 2).

## 4. Discussion

The aim of the current studies was to conduct a head-to-head comparison of the antinociceptive properties of morphine with or without SJP-005 at different morphine dosages. SJP-005 appeared to influence the antinociceptive properties of morphine when inflammatory pain was assessed via hypersensitivity to tactile stimuli in the von Frey up-and-down test (Study 1). Figure 1 shows that, compared to morphine alone, the observed paw response was consistently at higher levels after co-administering SJP-005. This was confirmed by a statistically significant difference in Δ paw withdrawal response for the morphine 3 mg/kg bw and SJP-005 combination. 

Although these findings could point to possible antinociceptive properties of SJP-005, the Δ paw withdrawal response scores were not statistically significant for higher morphine dosages. Also, the intercept and slope of the trendlines presented in Figure 1 did not significantly differ. In Study 2, assessing centrally acting pain, no antinociceptive properties of SJP-005 were observed using the hot plate test. This finding contrasts against those of a previous study in mice that did find antinociceptive effects of ketotifen using a hot plate test [13]. Given the inconsistent results, more research is needed to determine whether stronger painful stimuli can be handled by the animals after co-administering SJP-005 with morphine as compared to morphine with placebo. These observations should evaluate the possibility of an opioid sparing effect (i.e., that less morphine would be needed to achieve the same antinociceptive effect when SJP-005 is co-administered).

In the treatment of opioid use disorder, it would be beneficial for a treatment to have antinociceptive properties. The current treatments, i.e., clonidine and lofexidine, exert limited analgesic effects. Clonidine is sometimes used for the treatment of neuropathic pain, but it is not commonly prescribed for the treatment of somatic pain [18,19]. Also, the use of clonidine is frequently accompanied by adverse effects such as dry mouth, drowsiness, dizziness, mood changes, sleep problems, and headache. Lofexidine, an α-2 adrenergic receptor agonist, has no effects on pain ratings during opioid withdrawal [20]. Also, a study in 19 women revealed that lofexidine was not effective for the treatment of chronic pelvic pain [21]. The absence of pronounced antinociceptive effects of SJP-005 thus makes it comparable to the treatments that are currently used or in development for opioid use disorder. The finding is also somewhat surprising as the antinociceptive properties of ibuprofen are well-documented. 

Analgesic effects have been reported for ibudilast, a nonselective phosphodiesterase inhibitor, which is currently in development for the treatment for opioid use disorder. Clinical trials in opioid-dependent human subjects demonstrated ibudilast’s analgesic effects using a cold pressor test [22,23]. However, a study in migraine patients revealed that 8 weeks of treatment with ibudilast had no effect on the frequency or severity of moderate to severe headache [24]. 

The PPAR-γ agonist pioglitazone is another drug that has been investigated in relation to opioid use disorder. Using the hot plate test in mice, pioglitazone did not show antinociceptive properties [25]. However, pioglitazone did attenuate tactile allodynia (shown via von Frey test) and thermal hyperalgesia in neuropathic pain models in mice and rats [26,27,28,29], demonstrating that PPAR-γ agonists have relevant antinociceptive properties in animals that should be further investigated in humans. Of interest for the treatment of opioid withdrawal, another study in a rat model of diarrhea-predominant irritable bowel syndrome found that pioglitazone significantly reduced defecation frequency, produced a shift from watery stool to hard stool, and increased nociceptive thresholds [30]. On the other hand, clinical trials in humans failed to demonstrate sufficient efficacy of pioglitazone on the oxycodone abuse liability of nondependent opioid users [31] and in the treatment of opioid use withdrawal. Together with the observations made with SJP-005, this encourages further research into the antinociceptive properties and potential opioid sparing properties of PPAR-γ agonists. Preferably, these studies will also include assessments of biomarkers to explore the involvement of immune cells and mediators in the action of SJP-005.

Taken together, the current findings suggest that SJP-005 might have antinociceptive properties, and they also suggest a possible opioid sparing effect. To obtain a better understanding of the possible analgesic properties of SJP-005, future clinical trials in humans should be conducted. These could, for example, utilize a cold pressor test to further evaluate the antinociceptive properties of SJP-005. Also, pain complaints and the possible use of adjuvant medication to treat pain should be monitored in studies testing the efficacy of SJP-005 for opioid use withdrawal.

## Figures and Tables

**Table 1:** Paw volume.

		Pre-Dose Paw Volume (mL)	Post-Dose Paw Volume (mL)	Edema (mL)
Group	Treatment	Mean (SE)	Mean (SE)	Mean (SE)
1	Morphine 3 mg/kg bw + placebo	2.1 (0.05)	3.2 (0.07)	1.1 (0.07)
2	Morphine 10 mg/kg bw + placebo	2.0 (0.03)	2.9 (0.06)	0.9 (0.06)
3	Morphine 30 mg/kg bw + placebo	2.1 (0.03)	3.0 (0.08)	0.9 (0.08)
4	Morphine 3 mg/kg bw + SJP-005	2.2 (0.04)	3.0 (0.07)	0.9 (0.07)
5	Morphine 10 mg/kg bw + SJP-005	2.2 (0.02)	3.1 (0.10)	0.8 (0.09)
6	Morphine 30 mg/kg bw + SJP-005	2.1 (0.05)	3.0 (0.08)	0.9 (0.05)
7	Placebo + SJP-005	2.1 (0.05)	3.2 (0.08)	1.1 (0.10)

Mean and standard error (SE) are shown. All differences between pre- and post-dosing for paw volume were statistically significant (*p* < 0.0001). No significant differences in edema were found among the seven groups. Ibuprofen and ketotifen (SJP-005) were orally administered one after the other, followed 90 min thereafter by a subcutaneous injection of morphine (Group 1–6) or saline (Group 7). Paw volume was assessed approximately 30 min after morphine administration. Abbreviations: bw = bodyweight.

**Table 2:** Paw withdrawal response.

		Pre-Dose Paw Withdrawal	Post-Dose Paw Withdrawal	Pre- vs. Post-Dose	Placebo vs. SJP-005
Group	Treatment	Mean (SE)	Mean (SE)	*p*-Value	*p*-Value
1	Morphine 3 mg/kg bw + placebo	18.0 (3.0)	27.2 (3.1)	0.025 *	0.179
2	Morphine 10 mg/kg bw + placebo	18.8 (5.1)	59.3 (3.3)	0.000 *	0.491
3	Morphine 30 mg/kg bw + placebo	22.2 (5.7)	73.9 (7.7)	0.000 *	1.000
4	Morphine 3 mg/kg bw + SJP-005	22.1 (3.4)	46.8 (3.9)	0.000 *	-
5	Morphine 10 mg/kg bw + SJP-005	17.0 (3.6)	74.1 (8.2)	0.000 *	-
6	Morphine 30 mg/kg bw + SJP-005	17.3 (4.2)	76.4 (7.1)	0.000 *	-
7	Placebo + SJP-005	18.3 (3.8)	11.1 (1.6)	0.068	-

Mean and standard error (SE) of the minimal mechanical force (g) required to elicit a paw withdrawal response in 50% of animals are shown for the pre- and post-dose paw withdrawal response. *p*-values are shown for the statistical comparisons of the corresponding morphine + placebo group versus the morphine + SJP-005 group. For the placebo versus SJP-005 comparison, paired comparisons were made between Group 1 and Group 4, between Group 2 and Group 5, and between Group 3 and Group 6. Statistically significant differences (*p* < 0.05) are indicated by *.

**Table 3:** Hot plate response.

		Week 1 (Morphine Only)			Week 2 (Morphine + SJP-005)	
Group	Morphine Dose	Pre-Dose	Post-Dose	% Analgesia	Post-Dose	% Analgesia
1	0 mg/kg bw	4.1 (0.3)	3.4 (0.3)	-	2.7 (0.3)	-
2	3 mg/kg bw	4.3 (0.3)	5.2 (0.6)	53	3.2 (0.4)	19
3	10 mg/kg bw	4.2 (0.4)	9.1 (0.9) *	168	5.4 (0.7)	100
4	30 mg/kg bw	3.9 (0.2)	30.0 (0.0) *	782	25.6 (2.9)*	848

Mean and standard error (SE) in seconds are shown for the pre- and post-dose hot plate response. Significant differences (*p* < 0.05) between treatments and placebo are indicated by *. No significant differences were observed between Week 1 and Week 2 assessments. Ibuprofen and ketotifen (SJP-005) were orally administered one after the other, followed approximately 90 min thereafter by a subcutaneous injection of saline (Group 1) or morphine (Groups 2–4).

## Data Availability

The data are available in Appendix A and Appendix B.

## Appendix A

**Table A1:** Individual Data, Study 1.

Assessment		Paw Withdrawal Response (g)		Paw Volume (mL)		
Group	Animal	Pre-Dose	Post-Dose	Pre-Dose	Post-Dose	Edema
Group 1: Morphine (3 mg/kg)	5401	6.7	10.3	1.89	2.91	1.02
	5402	37.3	37.3	2.10	3.04	0.94
	5403	12.0	37.3	1.98	3.18	1.20
	5404	22.3	37.3	2.07	3.08	1.01
	5405	9.0	26.0	2.08	3.42	1.34
	5406	10.3	37.3	2.25	3.34	1.09
	5407	26.0	22.3	2.23	2.89	0.66
	5408	15.0	16.3	2.25	3.52	1.27
	5409	22.3	26.0	2.36	3.19	0.82
	5410	18.7	22.3	2.14	3.30	1.16
Group 2: Morphine (10 mg/kg)	5411	16.3	60.0	1.81	2.59	0.78
	5412	18.7	48.7	1.94	3.22	1.28
	5413	12.7	60.0	2.03	2.81	0.78
	5414	10.3	60.0	2.06	2.80	0.74
	5415	6.0	60.0	2.08	2.89	0.81
	5416	5.3	73.3	2.03	2.66	0.64
	5417	60.0	60.0	1.97	2.82	0.84
	5418	12.7	60.0	2.06	3.12	1.07
	5419	30.0	73.3	1.99	3.07	1.09
	5420	16.3	37.3	2.14	2.89	0.75
Group 3: Morphine (30 mg/kg)	5421	18.7	48.7	2.11	3.04	0.92
	5422	18.7	73.3	2.14	3.33	1.20
	5423	48.7	60.0	2.09	3.12	1.03
	5424	7.3	37.3	1.94	2.77	0.83
	5425	3.3	100.0	2.03	3.03	1.00
	5426	18.7	60.0	2.26	2.85	0.59
	5427	12.0	100.0	2.11	2.89	0.79
	5428	15.0	60.0	2.17	2.83	0.66
	5429	60.0	100.0	2.19	3.36	1.17
	5430	19.3	100.0	2.08	2.52	0.43
Group 4: SJP-005 + Morphine (3 mg/kg)	5431	45.0	60.0	2.24	2.89	0.65
	5432	6.0	48.7	2.27	2.91	0.63
	5433	23.3	33.7	2.00	3.00	1.00
	5434	12.7	22.3	2.16	3.59	1.43
	5435	26.3	48.7	2.17	3.05	0.89
	5436	22.3	48.7	2.35	3.28	0.93
	5437	30.0	48.7	2.13	2.99	0.86
	5438	16.3	60.0	2.16	2.90	0.73
	5439	16.3	37.3	2.12	2.86	0.73
	5440	22.3	60.0	1.94	2.95	1.01
Group 5: SJP-005 + Morphine (10 mg/kg)	5441	16.3	100.0	2.10	2.94	0.84
	5442	7.3	73.3	2.23	2.80	0.57
	5443	15.7	100.0	2.31	2.73	0.43
	5444	16.3	48.7	2.14	2.77	0.63
	5445	45.0	73.3	2.14	2.79	0.65
	5446	18.7	26.0	2.33	3.79	1.46
	5447	15.0	60.0	2.26	3.17	0.91
	5448	8.3	100.0	2.23	3.24	1.00
	5449	4.7	60.0	2.15	3.24	1.09
	5450	22.3	100.0	2.24	3.08	0.84
Group 6: SJP-005 + Morphine (30 mg/kg)	5451	9.0	100.0	2.07	3.20	1.13
	5452	16.3	73.3	2.10	2.87	0.77
	5453	48.7	60.0	2.08	2.95	0.87
	5454	7.7	100.0	2.11	3.03	0.92
	5455	30.0	60.0	2.21	3.10	0.90
	5456	18.7	60.0	2.24	3.08	0.84
	5457	15.0	100.0	2.06	3.00	0.94
	5458	6.0	37.3	1.72	2.44	0.72
	5459	15.0	73.3	2.09	3.29	1.20
	5460	6.7	100.0	1.83	2.62	0.79
Group 7: SJP-005 + Placebo (saline 5 mL/kg)	5461	15.7	10.3	2.05	3.22	1.17
	5462	15.0	6.0	2.30	3.00	0.71
	5463	12.7	11.3	2.19	3.08	0.90
	5464	16.3	9.0	2.26	3.45	1.19
	5465	20.0	22.3	2.26	3.47	1.21
	5466	26.0	15.0	2.28	3.20	0.92
	5467	12.7	10.3	2.06	2.78	0.72
	5468	7.7	4.0	2.07	3.50	1.43
	5469	48.7	12.7	1.99	3.63	1.63
	5470	8.0	9.7	1.86	3.15	1.29

## Appendix B

**Table A2:** Individual Data, Study 2.

Assessment		Reaction Time (Seconds)		
Treatment		+ Placebo (Week 1)		+ SJP-005 (Week 2)
Group	Animal	Baseline	30 Min	30 Min
Group 1: Placebo (saline 5 mL/kg)	4401	4.6	2.6	2.6
	4402	4.6	3.6	3.0
	4403	1.4	3.2	4.0
	4404	3.8	3.2	3.9
	4405	4.7	5.3	3.9
	4406	3.9	1.8	1.4
	4407	3.7	2.4	2.2
	4408	5.2	4.6	1.8
	4409	3.7	4.0	2.4
	4410	4.8	3.5	2.2
Group 2: Morphine (3 mg/kg)	4411	3.6	3.9	2.3
	4412	4.1	5.5	3.1
	4413	4.0	8.6	3.0
	4414	6.1	4.2	3.9
	4415	4.4	4.5	5.2
	4416	4.9	5.6	2.4
	4417	3.1	4.0	3.5
	4418	4.2	3.7	2.7
	4419	3.7	3.2	1.4
	4420	4.5	8.4	4.8
Group 3: Morphine (10 mg/kg)	4421	3.4	12.8	6.4
	4422	3.3	9.0	2.5
	4423	4.5	6.2	3.0
	4424	4.1	11.4	6.2
	4425	3.3	10.2	4.0
	4426	5.9	7.0	9.9
	4427	3.4	6.7	6.1
	4428	3.7	8.3	2.9
	4429	6.7	5.4	5.8
	4430	4.1	14.1	6.7
Group 4: Morphine (30 mg/kg)	4431	4.5	30.0	10.6
	4432	4.4	30.0	30.0
	4433	4.6	30.0	30.0
	4434	4.4	30.0	30.0
	4435	3.2	30.0	30.0
	4436	3.4	30.0	-
	4437	2.8	30.0	30.0
	4438	3.2	30.0	9.5
	4439	4.2	30.0	30.0
	4440	4.3	30.0	30.0
